# Retraction Note: Metformin-repressed miR-381-YAP-snail axis activity disrupts NSCLC growth and metastasis

**DOI:** 10.1186/s13046-025-03331-9

**Published:** 2025-02-27

**Authors:** Dan Jin, Jiwei Guo, Yan Wu, Weiwei Chen, Jing Du, Lijuan Yang, Xiaohong Wang, Kaikai Gong, Juanjuan Dai, Shuang Miao, Xuelin Li, Guoming Su

**Affiliations:** 1 Clinical Medicine Laboratory, Binzhou Medical University Hospital, Binzhou, 256603 People’s Republic of China; 2 Cancer research institute, Binzhou Medical University Hospital, Binzhou, 256603 People’s Republic of China; 3 Department of Thyroid and Breast Surgery, Binzhou Medical University Hospital, Binzhou, 256603 People’s Republic of China; 4 Department of Nursing, Binzhou Polytechnic University, Binzhou, 256603 People’s Republic of China

**Retraction Note:** ***J Exp Clin Cancer Res*** **39, 6 (2020)**

10.1186/s13046-019-1503-6

The Editor in Chief has retracted this article. After publication, a number of serious image integrity issues were raised, including but not limited to:

- numerous overlap in gels and blots which are meant to represent different conditions or proteins.
- undeclared splicing in figure 1M.
- repetitions within an image representing histological tissues in Figure 9G.
- partial overlap between panels in supplementary figures 3E and 4D.

The authors were not able to provide an acceptable explanation. Therefore the Editor has lost confidence in the data and the conclusions of this article.

Author Jiwei Guo disagrees with this retraction. All other authors did not respond to correspondence from the publisher regarding this retraction.

